# Alzheimer’s Disease-Associated Alternative Splicing of *CD33* Is Regulated by the HNRNPA Family Proteins

**DOI:** 10.3390/cells12040602

**Published:** 2023-02-13

**Authors:** Riho Komuro, Yuka Honda, Motoaki Yanaizu, Masami Nagahama, Yoshihiro Kino

**Affiliations:** 1 Department of Bioinformatics and Molecular Neuropathology, Meiji Pharmaceutical University, 2-522-1, Noshio, Kiyose-shi 204-8588, Japan; 2 Department of RNA Pathobiology and Therapeutics, Meiji Pharmaceutical University, 2-522-1, Noshio, Kiyose-shi 204-8588, Japan; 3 Department of Molecular and Cellular Biochemistry, Meiji Pharmaceutical University, 2-522-1, Noshio, Kiyose-shi 204-8588, Japan

**Keywords:** Alzheimer’s disease, CD33, HNRNPA1, alternative splicing

## Abstract

Genetic variations of *CD33* have been implicated as a susceptibility factor of Alzheimer’s disease (AD). A polymorphism on exon 2 of *CD33*, rs12459419, affects the alternative splicing of this exon. The minor allele is associated with a reduced risk of AD and promotes the skipping of exon 2 to produce a shorter CD33 isoform lacking the extracellular ligand-binding domain, leading to decreased suppressive signaling on microglial activity. Therefore, factors that regulate the splicing of exon 2 may alter the disease-associated properties of CD33. Herein, we sought to identify the regulatory proteins of CD33 splicing. Using a panel of RNA-binding proteins and a human CD33 minigene, we found that exon 2 skipping of *CD33* was promoted by HNRNPA1. Although the knockdown of HNRNPA1 alone did not reduce exon 2 skipping, simultaneous knockdown of HNRNPA1 together with that of HNRNPA2B1 and HNRNPA3 promoted exon 2 inclusion, suggesting functional redundancy among HNRNPA proteins. Similar redundant regulation by HNRNPA proteins was observed in endogenous *CD33* of THP-1 and human microglia-like cells. Although mouse *Cd33* showed a unique splicing pattern of exon 2, we confirmed that HNRNPA1 promoted the skipping of this exon. Collectively, our results revealed novel regulatory relationships between CD33 and HNRNPA proteins.

## 1. Introduction

Alzheimer’s disease (AD) is the leading cause of dementia in the elderly and is a detrimental neurodegenerative disease characterized by the pathological accumulation of extracellular amyloid beta (Aβ) plaques and intraneuronal neurofibrillary tangles [1]. Genetic studies have identified multiple genes that increase or decrease the susceptibility to late-onset AD [2]. These studies revealed that a number of AD risk genes are preferentially expressed in microglia [3], which are involved in various biological functions, including phagocytosis, synaptic pruning, and cytokine release [4,5,6]. CD33 is a transmembrane immune receptor that is highly expressed on microglia and macrophages and that binds to sialic acids, which are found in glycoproteins and glycolipids, via an extracellular domain [7]. CD33 contains an immunoreceptor tyrosine-based inhibitory motif (ITIM) and an ITIM-like sequence in the C-terminus [8]. The ITIM of human CD33 mediates inhibitory signals to restrict immune responses [9] but is absent in mouse CD33, whereas the ITIM-like motif is conserved between humans and mice. CD33 expression is increased in human AD brains and correlated with plaque burden as well as insoluble Aβ42 levels [10].

Initially, genome-wide association studies identified a single nucleotide polymorphism in the promoter region of the *CD33* gene, rs3865444, whose minor allele is associated with a modestly protective effect on AD susceptibility and reduced CD33 expression [10,11,12]. CD33 inhibits the uptake of Aβ by microglia, and mice lacking *Cd33* showed a reduced burden of amyloid plaques and insoluble Aβ42 [10,13]. Subsequently, rs12459419, a single-nucleotide polymorphism (SNP) located at exon 2 of *CD33*, was proposed as the causal variant that is in complete linkage disequilibrium with rs3865444, the aforementioned lead SNP [14,15]. rs12459419 is located in the coding region and results in an amino acid substitution (A14V). However, the main effect of this variant is thought to be alternative splicing of exon 2, which results in the production of a CD33 isoform lacking the extracellular IgV domain responsible for sialic acid binding. The minor T allele of rs12459419 is associated with increased exon 2 skipping as well as a reduced susceptibility to AD compared with the major C allele, named the common variant (CV). Importantly, the longer CD33 protein isoform containing exon 2 (CD33M) restrains microglia phagocytosis and, therefore, Aβ uptake, whereas the shorter isoform lacking exon 2 (CD33m) enhances phagocytosis [16,17]. In addition, the deletion of *CD33* enhances the secretion of cytokines in both human macrophage-like cells (THP-1) and microglia [18]. Thus, CD33 has been recognized as a key factor of microglial activity as well as the risk of AD, highlighting the regulation of exon 2 splicing as the key determinant of CD33 activity. Indeed, CD33 has become a potential therapeutic target for AD [19,20]. These findings suggest that certain RNA-binding proteins (RBPs) that regulate the alternative splicing of *CD33* exon 2 influence the pathogenesis of AD, and possibly other neurodegenerative diseases, through modulating microglial activity. Currently, only a few RBPs have been implicated in the splicing regulation of CD33 [21].

In this study, we sought to identify novel regulators of *CD33* splicing at exon 2. Using a CD33 minigene, we screened 22 RBPs and identified HNRNPA1 as a candidate. We verified that HNRNPA1 and associated paralogs, HNRNPA2B1 and HNRNPA3, repress exon 2 inclusion in a redundant manner, independently of the variation of rs12459419. This regulation is partly shared between human and mouse *CD33* orthologs. We also found novel splicing patterns in mouse *Cd33*. Thus, our results provide a novel molecular link between CD33 and HNRNPA proteins.

## 2. Materials and Methods

### 2.1. Plasmids

**Figure 1:**
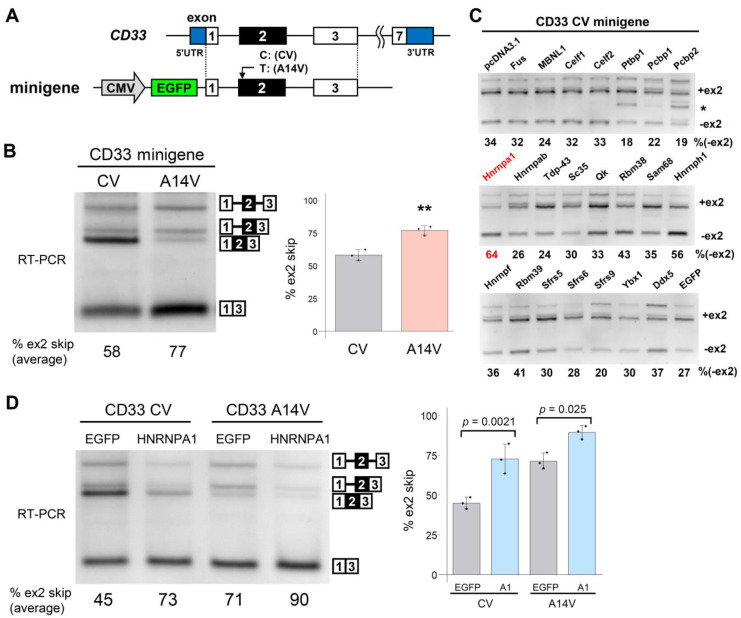
Screening of RNA-binding proteins (RBPs) that regulate the alternative splicing of *CD33* exon 2. (**A**) Schematic diagram of the human CD33 minigene. (**B**) Splicing assay of the CD33 minigenes (CV and A14V). A representative agarose gel image of RT-PCR amplification products using the CD33 minigenes (**top**). The bar chart shows the proportion of exon 2 skipping. Error bars represent SD (*n* = 3). ** *p* = 0.0040, two-tailed Welch’s *t*-test. (**C**) Splicing assay results using the CD33 CV minigene and a panel of RBPs. RBPs were expressed as a fusion with EGFP. Splice products were detected by RT-PCR using agarose gels. The proportion of exon 2 skipping is indicated at the bottom of each lane. (**D**) Splicing regulation of CV and A14V minigenes by human HNRNPA1. The bar chart shows the proportion of exon 2 skipping. Error bars represent SD (*n* = 3). Statistical significance was evaluated by Tukey’s test.

To construct human CD33 minigenes, the *CD33* genomic fragment was amplified from the genomic DNA of HMO6 cells via nested PCR using two primer sets—i.e., nest-CD33-Fw and nest-CD33-int3-Rv for the first PCR and BglII-CD33-ex1-Fw and XhoI-CD33-ex3-Rv for the second PCR—together with KOD-plus-NEO (TOYOBO, Tokyo, Japan) and digested with BglII and XhoI and then ligated into the BglII/SalI site of pEGFP-C1 (Clontech, Mountain View, CA, USA). As the cloned *CD33* fragment contained the major allele (T) at rs12459419 (CV minigene), this nucleotide was substituted to C by PCR-mediated mutagenesis using two primers, CD33-A14V-Fw and CD33-A14V-Rv, in addition to the above-mentioned primer set, BglII-CD33-ex1-Fw and XhoI-CD33-ex3-Rv, as described previously [22] to obtain the A14V minigene. To investigate the conservation of alternative splicing of exon 2 of the CD33 gene, *Mus musculus Cd33* exon 1 to exon 3 was amplified from Neuro2a genomic DNA and then digested with BglII and XhoI and cloned into pEGFP-C1 vector as above. Human HNRNPA1 was amplified from a cDNA library of HEK293 cells and cloned into the BglII/SalI site of pEGFP-C1. Although cDNA fragments of HNRNPA2B1 and HNRNPA3 could be amplified, plasmids containing them could not be obtained by conventional cloning strategies. To address this, we used in vitro assembly and amplification of plasmids containing EGFP or EGFP-fused HNRNPA proteins. Briefly, two PCR amplified fragments, (i) oriC and (ii) CMV promoter fused with genes coding for EGFP, and HNRNPA family proteins, or LacZ, and SV40 polyA signal (amplified from pEGFP-C1 plasmids or ligation products containing each cDNA), were assembled into a plasmid and amplified using the OriCiro Cell Free Cloning System (OriCiro Genomics, Tokyo, Japan), according to the manufacturer’s instructions. To engineer an inducible vector, *HNRNPA1* was subcloned into PB-Tet-EGFP-Azu-Puro. The resulting vector (PB-Tet-EGFP-A1-Azu-Puro) constitutively expressed both the Azurite blue fluorescent protein and the puromycin resistance gene with inducible expression of EGFP-HNRNPA1 upon Dox treatment. Mouse *Hnrnpa1* was amplified from the FANTOM3 clone (E430035K14, provided by Dr. Hayashizaki at RIKEN) and inserted into pEGFP-C1. The primers used in this study are listed in Table S1. RBP constructs used in the initial screening (Figure 1C) were described previously [23]. All constructs were confirmed by sequencing.

### 2.2. Cell Culture

HEK293 cells (RCB1637, Riken BRC, Ibaraki, Japan), Neuro2a cells (#CCL-131, ATCC, Manassas, VA, USA), and RAW264.7 cells (#91062702, ECACC) were grown in Dulbecco’s modified Eagle medium supplemented with 10% fetal bovine serum (FBS) and 1% penicillin/streptomycin (Thermo Fisher Scientific, Waltham, MA, USA) at 37 °C in 5% CO_2_. THP-1 cells (RCB1189, Riken BRC) were maintained in RPMI medium supplemented with 10% FBS, 1% penicillin/streptomycin, and 1× GultaMAX (Thermo Fisher Scientific) at 37 °C in 5% CO_2_. Human iMG cells were derived from Cellartis Microglia (from ChiPSC12) Kit (Takara Bio, Shiga, Japan), which were cultured for 7 days according to the manufacturer’s protocol. We also used iCell Microglia 01279 (Fujifilm, Osaka, Japan) cultured in iCell Glial Base Medium containing associated supplements (M1034, M1036, M1037, and M1046; Fujifilm), as recommended by the supplier.

### 2.3. Cellular Splicing Assay

A cellular splicing assay was performed as previously described [23]. In brief, HEK293 cells were seeded onto 12-well plates coated with 0.1% *v*/*w* gelatin (Wako, Osaka, Japan) on the day before transfection. A total of 0.02 μg of the minigene expression vector and 0.48 μg of the EGFP-fused protein expression vector were transfected using Lipofectamine 2000 (Thermo Fisher Scientific). To introduce siRNA, THP-1 cells were seeded onto 6-well plates, treated with 100 ng/mL phorbol 12-myristate 13-acetate on the next day for 24 h, and then transfected with 30 pmol siRNAs (the sequences of siRNAs are listed in Table S1) per well with Lipofectamine RNAiMAX (Thermo Fisher Scientific) for 72 h. Total RNA was harvested using the NucleoSpin RNA kit with DNase treatment (Takara Bio). Reverse transcription (RT) was performed using Revertra Ace-α-(TOYOBO) with oligo dT and random hexamers as primers. The amount of RNA was quantified using NanoDrop (Thermo Fisher Scientific) and adjusted to the same concentration among the samples. RT-PCR was performed using the Blend-Taq-plus-(TOYOBO) and primer sets listed in Table S1. Electrophoresis was performed using agarose gels (Invitrogen and pH Japan, Carlsbad, CA, USA) or e-PAGEL polyacrylamide gels (ATTO, Tokyo, Japan), and gels were stained with ethidium bromide (Genesee Scientific Corporation, San Diego, CA, USA). The appropriate number of PCR cycles was determined by sampling at multiple cycles. Gel images were captured using Luminograph III (ATTO) and quantified using CS Analyzer (ATTO). Original gel images are shown in Figure S9.

### 2.4. SDS-PAGE and Western Blotting

SDS-PAGE and Western blotting were performed as previously described [24]. Gel images were captured using Luminograph III (ATTO) and quantified using CSAnalyzer (ATTO). The antibodies used in this study are listed in Table S2. Original gel images are shown in Figure S9.

### 2.5. Ribonucleoprotein Immunoprecipitation (RIP)

RIP was performed essentially as described previously [23]. Here, anti-HNRNPA1 antibody (4B10, Biolegend, San Diego, CA, USA) and Dynabeads protein G (Thermo Fisher Scientific) were conjugated at the ratio of 4.2 μg of antibodies to 25 μL of beads. For quantitative PCR, two regions of *CD33* pre-mRNA, corresponding to a portion of either intron 2 or exon 7, were amplified. The amount of cDNA from the immunoprecipitated fractions was divided by that of the input fraction and multiplied by 100 (shown as % input).

### 2.6. Statistical Analysis

All graphs were produced using R (version 3.6.1, https://www.r-project.org/, accessed on 5 July 2019). EZR [25] was used in all cases to conduct statistical analyses. Error bars in all graphs represent standard deviations (SD). In bar charts, black dots indicate individual data points. Data were analyzed using two-tailed Welch’s *t*-test for comparison of two groups and Tukey’s test for comparisons of more than two groups. The statistical tests used are described in each figure legend.

### 2.7. Animals

This study was performed in accordance with the institutional guidelines and protocols approved by the Meiji Pharmaceutical University Committee for Ethics of Experimentation and Animal Care (approval number: 2901). C57BL/6J mice were purchased from Japan SLC and were maintained on a 12 h light/dark cycle with access to food and water *ad libitum*. The temperature and humidity were maintained at 23 °C ± 2 °C and 50% ± 10%, respectively. Animal health was checked by the animal facility staff twice a week. Female and male mice (three animals per group) were used for experiments at 8, 24, and 48 weeks.

## 3. Results

### 3.1. Screening of RBPs for the Regulators of CD33 Exon 2 Splicing

We first engineered minigenes covering the genomic region of human *CD33* exon 1 to exon 3 with major (C) or minor (T) alleles at rs12459419 (CV and A14V, Figure 1A). On transfection into HEK293 cells, CV showed the expected higher level of exon 2 inclusion than that of A14V (Figure 1B). Thus, our minigenes recapitulated the known effect of the AD-associated SNP. HEK293 cells do not express endogenous CD33 at a detectable level and, therefore, the CD33 signals detected after transfection should be primarily from the transgene. Several splicing products retained intron 1 [26] or both intron 1 and 2, together with exon 2 inclusion (Figure 1B). We regarded all bands containing exon 2 with or without intron retention as an exon 2 inclusion in the subsequent quantification. To identify the factors regulating the alternative splicing of *CD33* exon 2, we examined 22 RBPs derived from murine cDNA (except for MBNL1 of human origin) that were expressed as EGFP-fused proteins. We recently used this set of RBPs for screening splicing regulators of *TREM2* [23]. The EGFP-fused RBPs were transfected into HEK293 cells with the CV CD33 minigene. The splicing pattern of *CD33* exon 2 was determined by reverse transcription-polymerase chain reaction (RT-PCR) (Figure 1C). Transfection of an empty vector (pcDNA3.1) and EGFP served as negative controls. Among several RBPs that modified the splicing pattern of *CD33*, Hnrnpa1 promoted the strongest exon 2 skipping (Figure 1C). Therefore, we subsequently focused on the HNRNPA protein family in this study. We also noticed a band lacking the first 194 nucleotides of exon 3 (indicated by an asterisk, Figure 1C) when Ptbp1 or Pcbp2 was overexpressed. This was a novel splicing pattern using a cryptic 3′ splice site in exon 3 (Figure S1). As the screening was completed using murine cDNA constructs, we examined whether the human HNRNPA1 regulates the CD33 minigenes. HNRNPA1 promoted exon 2 skipping of both CV and A14V minigenes (Figure 1D), suggesting that the regulation by HNRNPA1 is independent of rs12459419.

### 3.2. The HNRNPA Family Proteins Redundantly Regulate the Splicing of CD33 Exon 2

**Figure 2:**
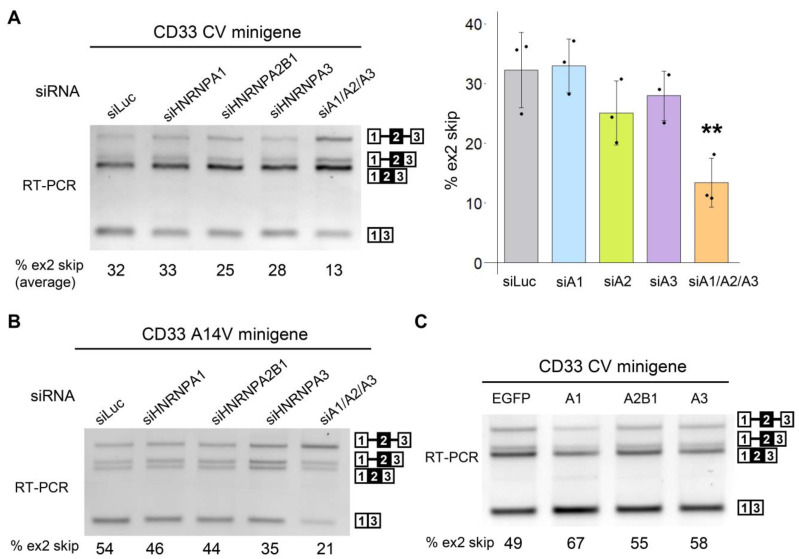
Alternative splicing of CD33 exon 2 is regulated redundantly by the HNRNPA family proteins. (**A**) RT-PCR products of the CD33 CV minigene in HEK cells treated with siRNA were resolved with agarose gel electrophoresis (**left panel**). The bar chart shows the portion of exon 2 skipping (**right panel**). Error bars represent the SD (*n* = 3). Tukey’s test was used for statistical evaluation (** *p* = 0.0063, in comparison with siLuc). (**B**) HEK cells were transfected with the A14V minigene and siRNA against the HNRNPA family and were analyzed in the splicing assay. (**C**) HEK cells were transfected with EGFP or EGFP-fused HNRNPA proteins and were analyzed in the splicing assay.

To confirm whether endogenous HNRNPA1 regulates the CD33 minigene, we introduced small interfering RNA (siRNA) targeting *HNRNPA1* and its paralogs (*HNRNPA2B1* and *HNRNPA3*) together with the CV minigene into HEK293 cells. RNA interference (RNAi) efficacy was confirmed via Western blot analysis (Figure S2A). Although exon 2 skipping was not altered when *HNRNPA1* or a paralog was individually silenced by RNAi, simultaneous knockdown of these three proteins significantly decreased exon 2 skipping (siA1/A2/A3, Figure 2A). Skipping of exon 2 in the A14V minigene was also decreased by the knockdown of endogenous *HNRNPA* paralogs (Figure 2B). We then tried to examine the effect of their overexpression. We PCR amplified the cDNA of human *HNRNPA2B1* and *HNRNPA3* but were unable to obtain plasmids containing these fragments by conventional cloning methods using *E. coli*. Therefore, we engineered plasmids containing EGFP-fused HNRNPA proteins using the OriCiro system that allowed enzymatic recombination and amplification of DNA fragments. Overexpression of each HNRNPA paralog promoted exon 2 skipping of the CD33 minigene (Figure 2C and Figure S2B). These results indicated that HNRNPA proteins redundantly regulate *CD33* splicing and, therefore, the effect of depleting one of the HNRNPA paralogs was masked by the others.

**Figure 3:**
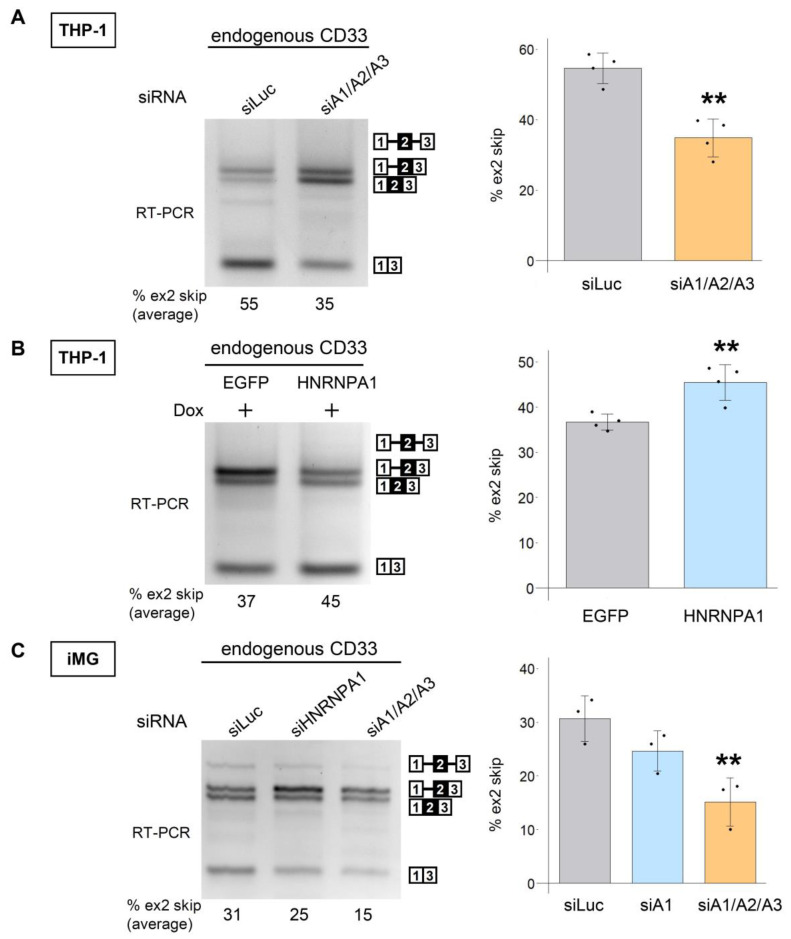
Endogenous *CD33* splicing is regulated by HNRNPA proteins. (**A**) THP-1 cells were treated with siRNA against luciferase or *HNRNPA* paralogs (siA1/A2/A3) and were assessed via splicing analysis (**left panel**). Quantified results are shown in the bar chart (*n* = 4). ** *p* = 0.0012 (two-tailed unpaired *t*-test). (**B**) Expression of EGFP or EGFP-HNRNPA1 in THP-1 cells was induced with 1 μg/mL doxycycline, and mRNA was amplified using RT-PCR to detect the splicing pattern of endogenous *CD33* (**left**). The bar chart shows the proportion of exon 2 skipping. Error bars represent SD (*n* = 4). Welch’s *t*-test was used for statistical evaluation (** *p* = 0.0066). (**C**) Microglia-like cells converted from human-induced pluripotent stem cell-derived microglial cells (iMG cells, Cellartis Microglia) were treated with siRNA, and mRNA was amplified using RT-PCR (**left panel**), which was quantitatively analyzed (**right panel**). Error bars represent the SD (*n* = 3). Tukey’s test was used for statistical evaluation (** *p* = 0.0091).

We next examined the effect of HNRNPA proteins on the splicing of endogenous *CD33*. Simultaneous knockdown of HNRNPA proteins decreased exon 2 skipping of *CD33* in THP-1 cells (Figure 3A). As the very low transfection efficiency of THP-1 precluded experiments based on transient overexpression, we made an inducible THP-1 cell line that expressed EGFP or EGFP-HNRNPA1 upon doxycycline (Dox) treatment (Figure S3). Dox induction of EGFP-HNRNPA1 increased exon 2 skipping of endogenous *CD33* in comparison with that of EGFP alone (Figure 3B). Finally, we tested the splicing patterns of *CD33* in induced pluripotent stem cell (iPSC)-derived microglial cells (iMG cells). Consistent with the preceding results, simultaneous knockdown of HNRNPA proteins, but not single knockdown of HNRNPA1, decreased exon 2 skipping (Figure 3C). To confirm the reproducibility of these results, we used additional human iMG cells from a different supplier (iCell Microglia). As expected, we observed a reduction in exon 2 skipping via the knockdown of HNRNPA paralogs in these cells (Figure S4). Collectively, we confirmed that HNRNPA proteins regulate human *CD33* splicing.

**Figure 4:**
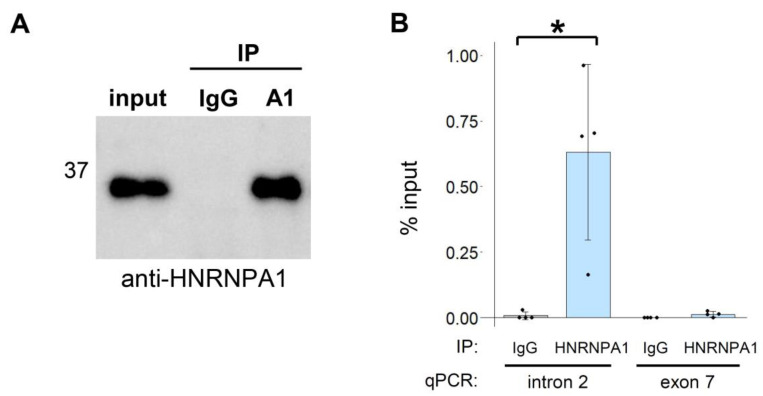
Ribonucleoprotein immunoprecipitation (RIP) of HNRNPA1 and *CD33*. (**A**) Immunoprecipitation of HNRNPA1 in THP-1 cells using an anti-HNRNPA1 antibody. (**B**) Two regions (intron 2 and exon 7) of *CD33* were amplified by RT-PCR using RNA in anti-HNRNPA1 antibody or IgG immunoprecipitates. The bar chart shows the results of the quantitative PCR analysis of *CD33* (*n* = 4). Error bars represent SD. * *p* = 0.034 (Welch’s *t*-test).

Lastly, we examined the intracellular association between HNRNPA1 and *CD33* pre-mRNA by ribonucleoprotein immunoprecipitation. RNA was extracted from anti-HNRNPA1 or IgG immunoprecipitates and amplified via RT-qPCR. A region of intron 2, but not exon 7, was significantly enriched by anti-HNRNPA1 compared with that of IgG (Figure 4A,B), suggesting that HNRNPA1 directly binds to the pre-mRNA of *CD33* in the vicinity of the regulated exon.

### 3.3. Conserved and Unique Features of Mouse Cd33 Splicing

**Figure 5:**
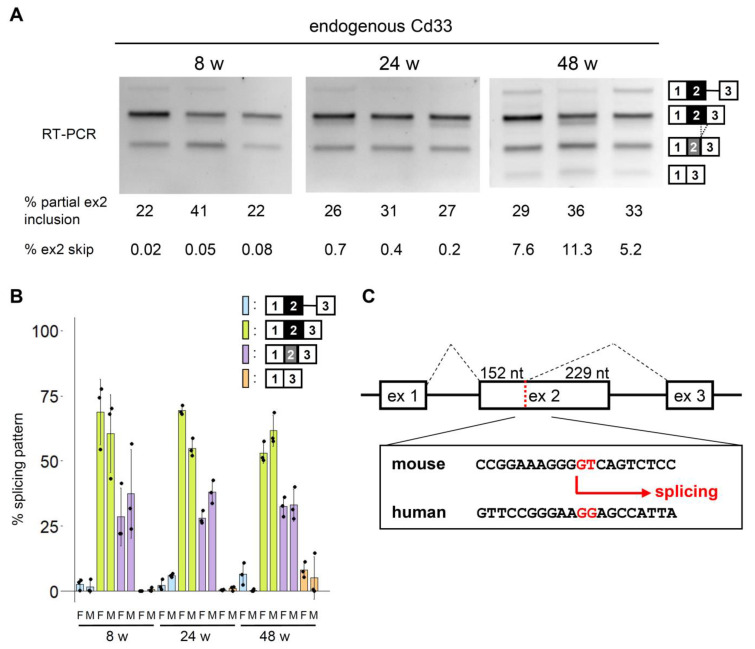
Splicing of *Cd33* in mouse brain. (**A**) Splicing assay of mouse *Cd33* in the hippocampus of female mice of different ages. (**B**) Quantification of the splicing patterns of *Cd33* in female and male mice. Error bars represent SD (*n* = 3). (**C**) Comparison of the exon 2 sequences of human and mouse *CD33* genes. Mouse *Cd33* contains a GT dinucleotide that constitutes a cryptic 5′ splice site.

We next investigated the conservation of the alternative splicing of the *CD33* exon 2. Splicing of murine endogenous *Cd33* was analyzed using mouse hippocampi of different ages. Exon 2 skipping was barely detectable at 8 and 24 weeks but could be detected at 48 weeks (Figure 5A,B and Figure S5A). We noticed a mouse-specific splicing pattern, in which exon 2 was partially included, as the second most dense band (Figure 5A). This product lacked a 3′ portion of exon 2 (229 nucleotides) because of the presence of a GT dinucleotide that acts as a cryptic 5′ splice site and is not conserved in human *CD33* (Figure 5C). We also analyzed the splicing of *Cd33* exon 2 using RNA-seq data available from GEO DataSets (Figure S5B–D, Table S3). Since RNA-seq data from brain tissues such as the hippocampus, as well as single-cell RNA-seq data, contained a small number of *Cd33* reads insufficient for splicing analysis, we focused on bulk RNA-seq of isolated cells such as microglia from brain tissues (Figure S5B,C). Consistent with our results, partial exon 2 inclusion was commonly observed in different cell types, accounting for ~20% of splicing products, with an exception of monocytes showing a relatively smaller proportion (~10%) of this pattern (Figure S5B–D). Exon 2 skipping was rare in general but was largely cell type-dependent, with the highest proportion of this pattern shown in mast cells (Figure S5D).

**Figure 6:**
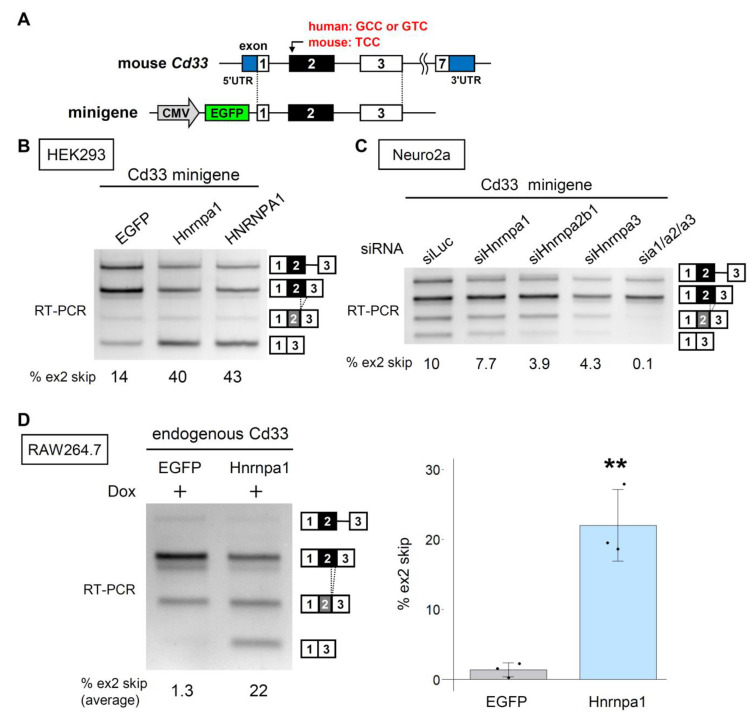
Splicing of mouse *Cd33* is regulated by HNRNPA family proteins. (**A**) Schematic diagram of the mouse Cd33 minigene. The sequence of the codon overlapping with rs12459419 in human *CD33* and the mouse counterpart are indicated in red. (**B**) Splicing assay of mouse Cd33 minigene in HEK293 cells. Quantification of exon 2 skipping (the lowest band) is shown at the bottom. (**C**) Splicing assay of mouse Cd33 minigene in Neuro2a cells treated with the indicated siRNA(s). Quantification is as in B. (**D**) Expression of EGFP or EGFP-Hnrnpa1 in RAW264.7 cell was induced with 1 μg/mL doxycycline and assessed in the splicing assay. The bar chart shows the ratio of exon 2 skipping (*n* = 3). Error bars represent SD. ** *p* = 0.0024 in a two-tailed Welch’s *t*-test.

We then constructed a minigene covering a genomic sequence from exon 1 to exon 3 of mouse *Cd33* (Figure 6A), to examine whether the splicing of exon 2 in mouse *Cd33* is regulated by the HNRNPA proteins. Similar to the human minigenes, the mouse Cd33 minigene showed alternative splicing of exon 2, which was promoted by the expression of human and mouse HNRNPA1 in HEK293 cells (Figure 6B). Moreover, RNAi-mediated knockdown revealed that exon 2 splicing was altered by the triple knockdown of murine HNRNPA family proteins in Neuro2a cells (Figure 6C). As RAW264.7 macrophage-like cells showed no basal exon 2 skipping of endogenous *Cd33*, we could not evaluate the further reduction in exon 2 skipping by the knockdown of HNRNPA proteins in this cell line (Figures S2C and S6).

We then established a RAW264.7 cell line that inducibly expresses EGFP or EGFP-Hnrnpa1 (murine) (Figure S7). Upon induction of EGFP-Hnrnpa1, exon 2 skipping of endogenous *Cd33* was clearly observed (Figure 6D). These results indicate that exon 2 of mouse *Cd33* is regulated by murine HNRNPA proteins as in human *CD33* while showing a mouse-specific splicing pattern.

## 4. Discussion

In this study, we investigated the regulatory proteins for *CD33* splicing and found that HNRNPA proteins promote exon 2 skipping in a redundant manner. Identification of RBPs regulating *CD33* exon 2 may provide novel therapeutic avenues to protect against AD. Recently, another study conducted a siRNA screening using a CD33 minigene system and identified SRSF1 and PTBP1 as splicing regulators of exon 2 [21]. In that study, HNRNPA1 was evaluated in the siRNA screening but was not detected as a regulator. This is consistent with our current results showing that single knockdown of HNRNPA1 did not significantly affect exon 2 splicing because of the presence of HNRNPA2B1 and HNRNPA3 (Figure 2). In contrast, overexpression of HNRNPA1 alone was sufficient to enhance exon 2 skipping (Figure 1C,D), which identified splicing regulation by HNRNPA proteins. Thus, overexpression-based screening can complement siRNA screening by identifying candidate proteins whose effect is undetectable in RNAi-based screening because of redundant regulation or low endogenous expression. Our results indicated that the loss of one HNRNPA protein function can be substituted by the activity of the others, highlighting the importance of the simultaneous depletion of paralogs in clarifying the effect of HNRNPA proteins. Indeed, we observed up-regulation of HNRNPA1 when HNRNPA2B1 was depleted (Figure S2A). Moreover, Dox induction of EGFP-HNRNPA1 reduced the level of endogenous HNRNPA1 in both THP-1 and RAW264.7 cell lines (Figures S3C and S7C). Although the precise mechanism of the regulation of *CD33* by HNRNPA proteins remains elusive, our RIP assay suggested that these proteins directly bind to a certain region near exon 2 of *CD33* pre-mRNA.

Alternative splicing of mouse *Cd33* has been poorly characterized. In this study, we detected exon 2 skipping at relatively low levels in the mouse hippocampus. Murine exon 2 skipping increased with aging with some variability among animals (Figure 5 and Figure S5A); therefore, it is interesting to determine the pattern of exon 2 splicing in older mice and AD model mice. RNA-seq data suggested that microglia of AD model mice showed a similar pattern of *Cd33* splicing to that of control mice (Figure S5C) and that microglia of old mice showed a tendency of a higher level of partial exon 2 inclusion (Figure S5B). However, it will be crucial to analyze multiple ages and different mouse models in the future, using a more sensitive RT-PCR assay in order to address this question. As with human *CD33*, exon skipping of mouse *Cd33* was promoted by murine HNRNPA proteins, indicating the evolutionary conservation of the regulatory relationship between the HNRNPA family and CD33. In addition, we identified partial exon 2 inclusion with an alternative 5′ splice site usage in *Cd33* (Figure 5C). This spliced product is predicted to produce a truncated protein or may experience nonsense-mediated mRNA decay due to frameshifting (Figure S8). We also detected intron retention in human and mouse *CD33* and partial exon 3 skipping in human *CD33*, all of which would result in the truncation of the C-terminus (Figure S8). Thus, CD33 protein expression is probably more extensively regulated at the level of pre-mRNA splicing than previously thought.

The HNRNPA family proteins are well-characterized RBPs involved in a number of aspects of RNA metabolism [27,28]. Interestingly, mutations of *HNRNPA1* and *HNRNPA2B1* are identified in families of multisystem proteinopathy or amyotrophic lateral sclerosis [29]. These proteins are also associated with pathological changes in neurological diseases [30,31,32]. Furthermore, HNRNPA1 is involved in Tau exon 10 splicing [33]. Therefore, the deregulation of HNRNPA proteins is of particular interest in the context of neurological diseases, though their contribution to the pathophysiology of microglia and macrophages has been elusive. Our current results propose HNRNPA proteins as potential regulators of microglial functions through *CD33* splicing, which warrants further investigations. Although a recent study has indicated several molecular differences between the human and mouse CD33 proteins [34], *Cd33* is involved in AD-related pathologies in mice [13]. Therefore, it would be interesting to determine whether murine HNRNPA proteins play a role in the regulation of *Cd33* as well as the activity of microglia in wild-type and AD model mice. One limitation of this study is the lack of functional analysis of HNRNPA proteins in microglia, which we will investigate in a future study. In conclusion, our results revealed that HNRNPA1 and associated paralogs are regulators of the alternative splicing of *CD33* exon 2 in different species.

## Data Availability

The datasets generated and/or analyzed during the current study are available from the corresponding author upon reasonable request.

## Supplementary Materials

The following supporting information can be downloaded at: https://www.mdpi.com/article/10.3390/cells12040602/s1, Figure S1: A cryptic splice site induced by EGFP-Ptbp1 and EGFP-Pcbp2; Figure S2: Expression of HNRNPA family proteins; Figure S3: A THP-1-based cell line inducibly expressing EGFP or EGFP-HNRNPA1; Figure S4: *CD33* splicing regulation by HNRNPA proteins in human microglial-like cells; Figure S5: Alternative splicing of mouse *Cd33* exon 2; Figure S6: Effect of siRNA targeting HNRNPA proteins on *Cd33* splicing in RAW264.7 cells; Figure S7: RAW264.7-based cells inducibly expressing EGFP or EGFP-Hnrnpa1; Figure S8: Prediction of CD33 proteins derived from the detected splicing products; Figure S9: Uncropped gel images; Table S1: List of oligonucleotides used in this study; Table S2: List of antibodies used in this study; Table S3: List of RNA-seq data used for splicing analysis.
