# Supplementary material for: Alzheimer’s Disease-Associated Alternative Splicing of CD33 Is Regulated by the HNRNPA Family Proteins
Source: Cells. 2023 Feb 13;12(4):602. doi: 10.3390/cells12040602 (PMC9954446; doi:10.3390/cells12040602)
Supplement: Supplementary file 1 [file cells-12-00602-s001.zip › cells-2002604-supplementary.pdf]

Supplementary information

Alzheimer's disease-associated alternative splicing of *CD33* is regulated by the HNRNPA family proteins

Riho Komuro[1,2,3], Yuka Honda[1], Motoaki Yanaizu[1,2], Masami Nagahama[3], Yoshihiro Kino[1,2,*]

[1]Department of Bioinformatics and Molecular Neuropathology, Meiji Pharmaceutical University, 2-522-1, Noshio, Kiyose-shi, Tokyo 204-8588, Japan

[2]Department of RNA Pathobiology and Therapeutics, Meiji Pharmaceutical University, 2-522-1, Noshio, Kiyose-shi, Tokyo 204-8588, Japan

[3]Department of Molecular and Cellular Biochemistry, Meiji Pharmaceutical University, 2-522-1, Noshio, Kiyose-shi, Tokyo 204-8588, Japan

[*]Corresponding author: Tel.: +81-42-495-8679, E-mail: kino@my-pharm.ac.jp

Supplementary Materials and Methods

Azurite-NLS vector

The Azurite variant of blue florescence protein was engineered from pEBFP-C1 (Takara Bio, Shiga, Japan) by PCR-mediated mutagenesis to introduce T66S, V151I, V225R, and L232R substitutions using primers including Azu-g197-Fw, Azu-g197-Rv, Azu-a451-Fw, Azu-a451-Rv, and BspEI-Azu-Rv according to a method previously described [1]. The fragment of Azurite was fused with three copies of the SV40 nuclear localization signal (encoding PKKKRKV) by insertion into pEGFP-C1-NLS vector [1], resulting in pAzurite-NLS.

Construction of the PB-Tet-Azu-Puro vector

PB-Cuo-MCS-IRES-GFP-EF1-CymR-Puro (System Biosciences, Palo Alto, CA, USA) was digested with NotI and SalI to remove a fragment covering EMCV-IRES, copGFP, EF1a promoter, and CymR-2A-Puro. The EF1a promoter and CymR-2A-Puro fragment was then PCR amplified using a primer set, NotI-EF1a-Fw and SalI-Puro-Rv, digested with NotI and SalI, and ligated into the digested vector. The Cumate Switch promoter was replaced with a CMV promoter using NheI and SpeI. Then, the

EF1a-CymR-2A-Puro fragment was replaced with EF1a-Azurite-NLS-2A-Puro that was assembled using NEBuilder HiFi DNA Assembly Master Mix (New England Biolabs, Ipswich, MA, USA) and three PCR fragments of EF1a, Azurite-NLS, and 2A-Puro. The resulting vector was designated as PB-CMV-Azu-Puro. The CMV promoter of this vector was replaced at the SpeI/SalI sites with a fragment containing Tet-ON-3G and Tet-3GS amplified from pAAV-TetOne vector (Takara Bio) using primer set NheI-Tet-Fw and SalI-Tet-Rv, resulting in the PB-Tet-Azu-Puro vector. EGFP, EGFP-HNRNPA1, and EGFP-Hnrnpa1 were inserted in the multi-cloning site downstream of the Tet3GS sequence in PB-Tet-Azu-Puro.

Inducible cell lines

To establish inducible EGFP-HNRNPA1 and EGFP cell lines, THP-1 cells were transfected with PB-Tet-EGFP-A1-Azu-Puro and PB-Tet-EGFP-Azu-Puro together with a plasmid encoding Super PiggyBac, which was subcloned from Super PiggyBac transposase expression vector (Systems Biosciences), using GeneXPlus (ATCC, Manassas VA, USA). EGFP-positive cells were selected with puromycin and then plated at 1 cell per well in a 96-well plates by fluorescence-activated cell sorting using a MA900 cell sorter (SONY, Tokyo, Japan). Cell clones were checked for their protein

expression upon doxycycline treatment (1 μg/ml). Similarly, RAW267.4-based cells were established using PB-Tet-Azu-Puro vectors containing EGFP or EGFP-Hnrnpa1 that were transfected using the K2 Transfection System (Biontex, Landsberger, München, Germany) according to the manufacturer's protocol. Cell pictures were obtained using EVOS M5000 Imaging System (Thermo Fisher Scientific, Waltham, MA, USA). Although Azurite-NLS is expressed under the EF1-alpha promoter, we repeatedly observed its up-regulation when dox was added, probably because Azu-NLS is also located downstream of the dox-inducible promoter (Tet-3GS).

Splicing analysis using RNA-seq data

RNA-seq data were retrieved at GEO DataSets (https://www.ncbi.nlm.nih.gov/gds). We chose datasets containing at least three experiments (three samples) for one condition or one cell type. Read counts corresponding to *Cd33* splicing patterns were determined using blastn with the setting of megablast as program, SRA as database, and the following junctional sequences as queries: "GCTGTCACTGCATGTGACCTCTCTATCACGGAC TCCTGAC" for conventional exon 2 inclusion, "GCTCCTGGCTCCGGA AAGGGCTCTATCACGGACTCCTGAC" for partial inclusion of exon 2, and "GCTGTTCTTGCTGTGT

GCAGCTCTATCACGGACTCCTGAC" for exon skipping. The number of sequence matched was regarded as the read count for each splicing pattern. The proportion of each splicing patterns in an experiment was calculated as the read count of a pattern divided by the sum of all patterns and then multiplied by 100. We then calculated the average and the standard deviation of the proportion of splicing patterns of a cell type or a condition.

Supplementary reference

1. Kino, Y., et al., *Nuclear localization of MBNL1: splicing-mediated autoregulation and repression of repeat-derived aberrant proteins.* Hum Mol Genet, 2015. **24**(3): p. 740-756.

Table S1. List of oligonucleotides used in this study.

Table S2. List of antibodies used in this study.

Table S3. List of RNA-seq data used for splicing analysis.

Figure S1. A cryptic splice site induced by EGFP-Ptbp1 and EGFP-Pcbp2.

Schematic illustration of cryptic 3 splice site in exon 3.

Figure S2. Expression of HNRNPA family proteins.

(**A**) HEK293 cells were treated with siRNA(s) and then analyzed with western blotting using indicated antibodies.

(**B**) HEK293 cells were transfected with indicated plasmids obtained from the OriCiro system. Cell lysates were analyzed with western blotting using an anti-GFP antibody.

(**C**) Neuro2a cells were treated with siRNA(s) and analyzed with western blotting using indicated antibodies.

Figure S3. A THP-1-based cell line inducibly expressing EGFP or EGFP-HNRNPA1.

(**A**) Structure of PB-Tet-Azu-Puro vector used for inducible expression of EGFP and EGFP-HNRNPA1. Azu: Azurite blue fluorescence protein fused with three copies of

nuclear localization signal. PB: PiggyBac; pA: SV40 polyA signal; hPGK: Tet-On 3G: Tet-On 3G transactivator, human PGK promoter; TB: transcription blocker; Tet-3GS: Tet-3GS promoter; EF1a: EF1a promoter; puro: puromycin resistance gene; ins: insulator; 2A: T2A self-cleaving peptide.

(**B**) Expression of Azu and EGFP-HNRNPA1 in the THP-1-based cells harboring the above minigenes. Cell images in the presence and absence of doxycycline are shown. Azu is visualized using DAPI filter panels. Scale bar: 150 μm.

(**C**) Expression of EGFP and EGFP-HNRNPA1 detected in a western blot analysis using indicated antibodies. As Azu is a variant protein derived from GFP, it cross-reacts with anti-GFP antibody.

Figure S4. *CD33* splicing regulation by HNRNPA proteins in human microglial-like cells.

Quantitative analysis (right panel) of human induced pluripotent stem cell-derived microglia-like cells (iCell Microglia) treated with siRNA and subjected to the *CD33* splicing assay using RT-PCR (left panel). Error bars represent the SD (n = 4). Welch's t-test was used for statistical evaluation (*p = 0.0102).

Figure S5. Alternative splicing of mouse *Cd33* exon 2.

(**A**) Splicing assay of mouse *Cd33* in the hippocampus of male mice of different ages.

(**B**) Splicing analysis of *Cd33* using RNA-seq data (GSE164675) of microglial cells isolated from 3 and 17 months old mice (n = 4).

(**C**) Splicing analysis of *Cd33* using RNA-seq data (GSE171195) of microglial cells isolated from transgenic Alzheimer's disease model male mice and non-transgenic control male mice at 10 months old (n = 3).

(**D**) Splicing analysis of *Cd33* using RNA-seq data of myeloid cells from PyMT breast cancer model mice (n = 3, female at unspecified age, GSE212643), dendritic cells (n = 4, from 6–8 weeks old female mice, GSE205069), and Mast Cells (n = 3, from bone mallow of BALB/c mice, GSE206630). In B-D, error bars represent SD.

Figure S6. Effect of siRNA targeting HNRNPA proteins on *Cd33* splicing in RAW264.7 cells.

(**A**) RAW264.7 cells were treated with siRNA and analyzed using *Cd33* splicing assay. There was negligible detection of exon 2 skipping.

(**B**) Quantification of splicing assay results. Bar chart shows the proportion of partial exon 2 inclusion (n = 3). Error bars represent SD. No significant difference was

observed (Tukey's test).

Figure S7. RAW264.7-based cells inducibly expressing EGFP or EGFP-Hnrnpa1.

(**A**) PB-Tet-Azu-Puro vector used for inducible expression of EGFP and EGFP-Hnrnpa1. Azu: Azurite blue fluorescence protein fused with three copies of nuclear localization signal. Structure details are as in Fig. S3A.

(**B**) Expression of Azu and EGFP-Hnrnpa1 in the RAW264.7 cells harboring the above constructs. Cell images in the presence and absence of doxycycline are shown. Azu was visualized using DAPI filter panels. Scale bar: 150 µm.

(**C**) Expression of EGFP and EGFP-Hnrnpa1 detected with western blotting using indicated antibodies.

Figure S8. Prediction of CD33 proteins derived from the detected splicing products. Termination codons that lead to truncation of the C-terminus are shown in red.

Figure S9. Uncropped gel images.

Figure S1

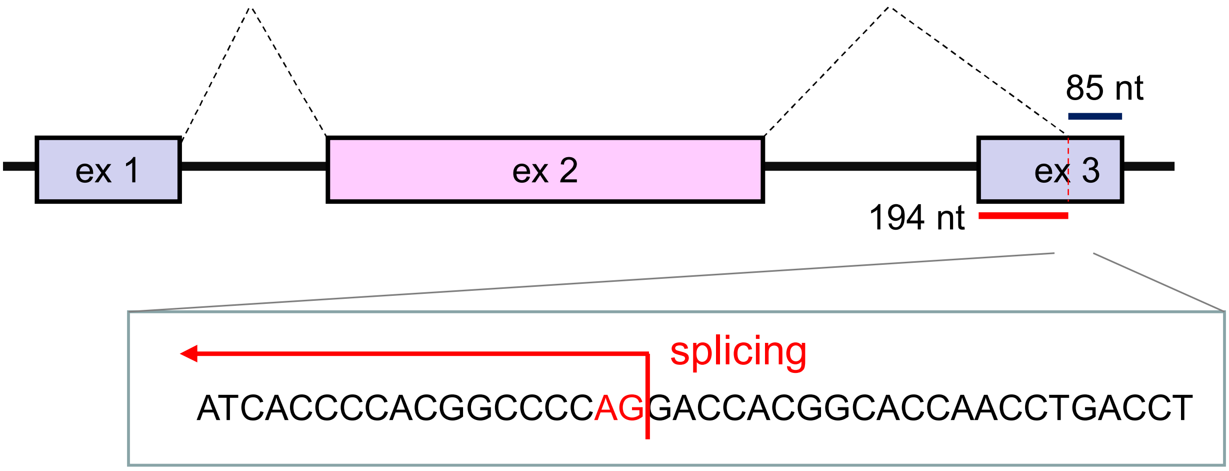

Figure S1. A cryptic splice site induced by EGFP-Ptbp1 and EGFP-Pcbp2.
Schematic illustration of cryptic 3 splice site in exon 3.

Figure S2

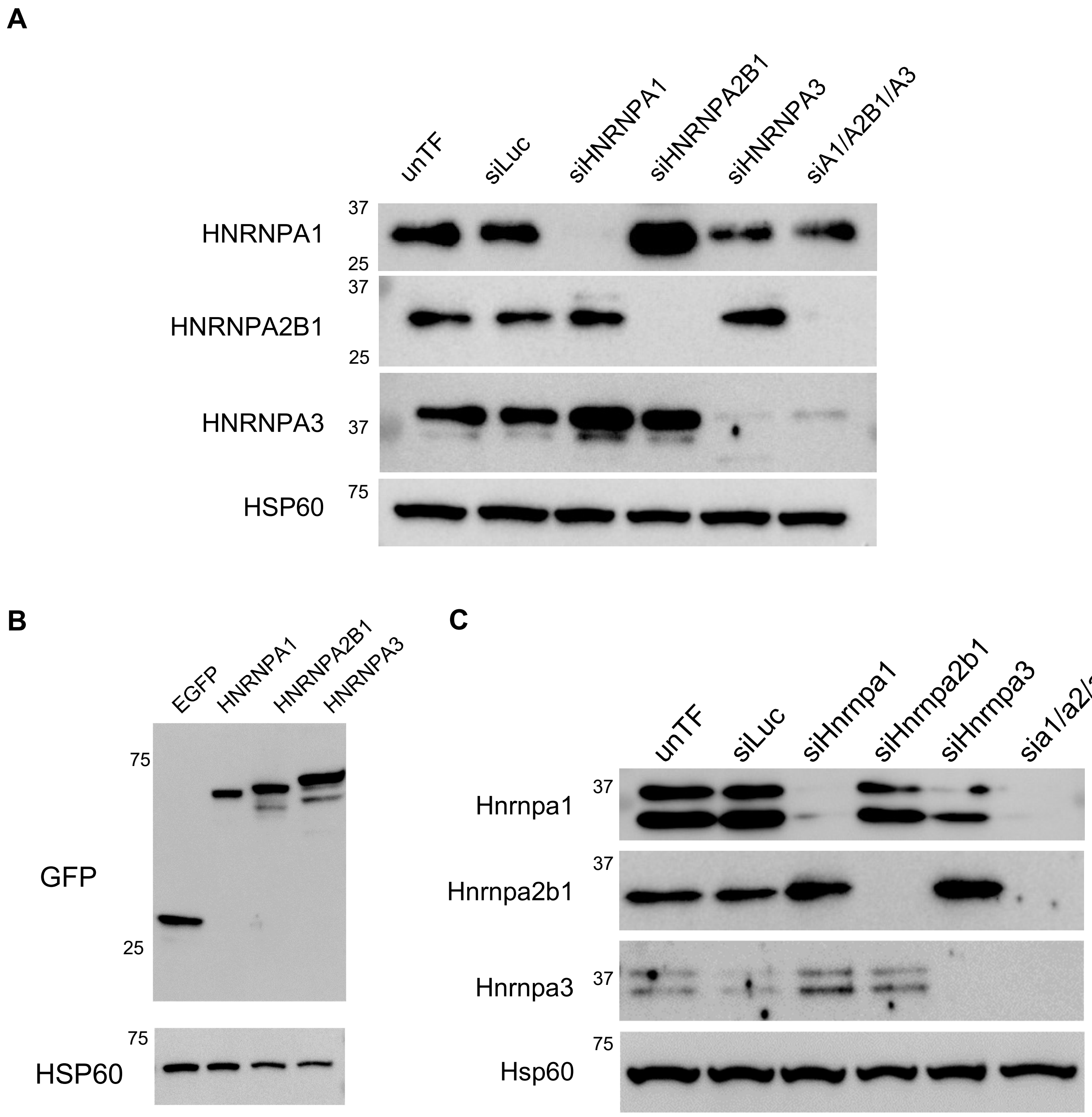

Figure S2. Expression of HNRNPA family proteins.

(**A**) HEK293 cells were treated with siRNA(s) and then analyzed with western blotting using indicated antibodies.

(**B**) HEK293 cells were transfected with indicated plasmids obtained from the OriCiro system. Cell lysates were analyzed with western blotting using an anti-GFP antibody.

(**C**) Neuro2a cells were treated with siRNA(s) and analyzed with western blotting using indicated antibodies.

Figure S3

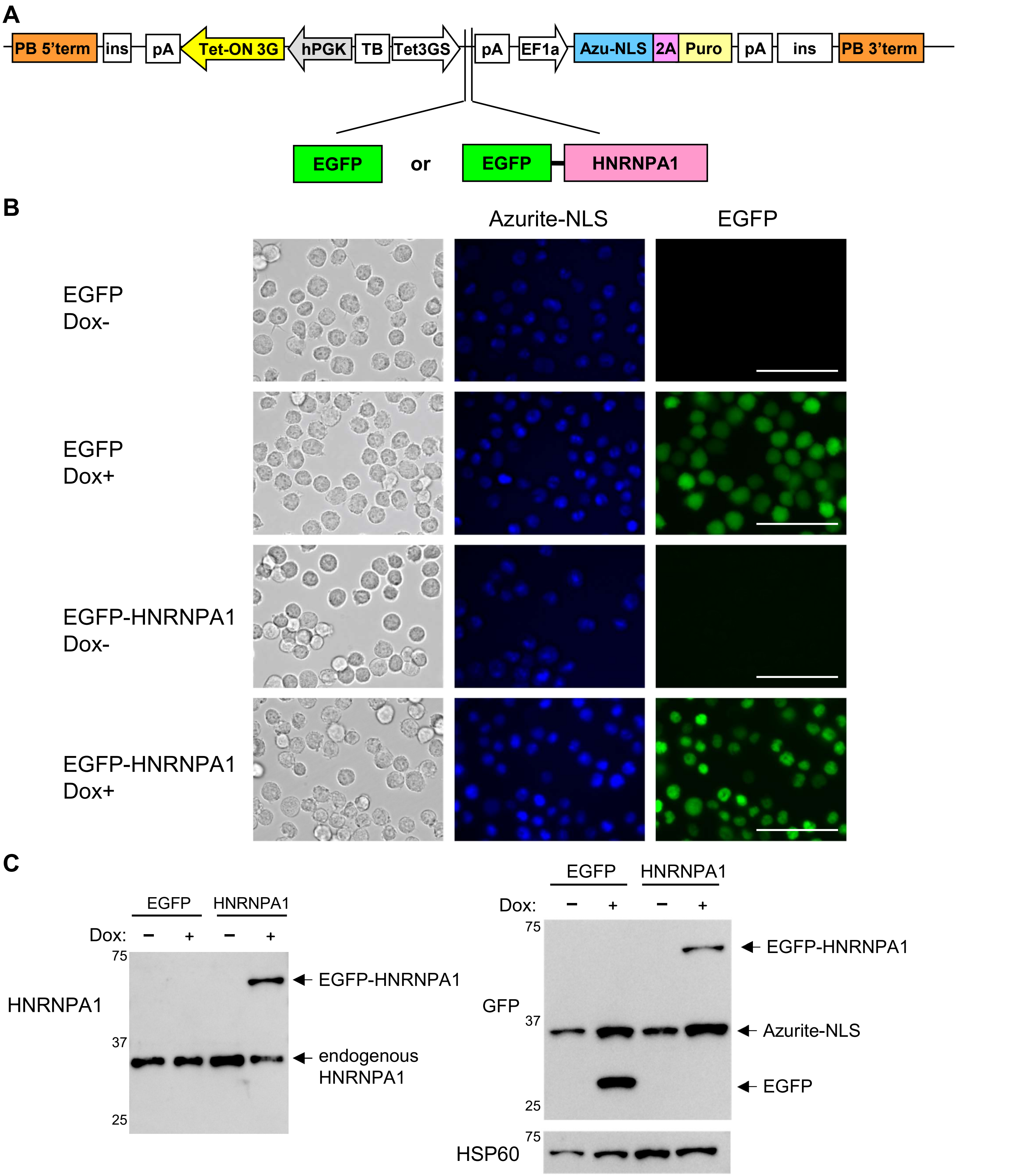

Figure S3. A THP-1-based cell line inducibly expressing EGFP or EGFP-HNRNPA1.
(**A**) Structure of PB-Tet-Azu-Puro vector used for inducible expression of EGFP and EGFP-HNRNPA1. Azu: Azurite blue fluorescence protein fused with three copies of nuclear localization signal. PB: PiggyBac; pA: SV40 polyA signal; hPGK: Tet-On 3G: Tet-On 3G transactivator, human PGK promoter; TB: transcription blocker; Tet-3GS: Tet-3GS promoter; EF1a: EF1a promoter; puro: puromycin resistance gene; ins: insulator; 2A: T2A self-cleaving peptide.
(**B**) Expression of Azu and EGFP-HNRNPA1 in the THP-1-based cells harboring the above minigenes. Cell images in the presence and absence of doxycycline are shown. Azu is visualized using DAPI filter panels. Scale bar: 150 μm.
(**C**) Expression of EGFP and EGFP-HNRNPA1 detected in a western blot analysis using indicated antibodies. As Azu is a variant protein derived from GFP, it cross-reacts with anti-GFP antibody.

Figure S4

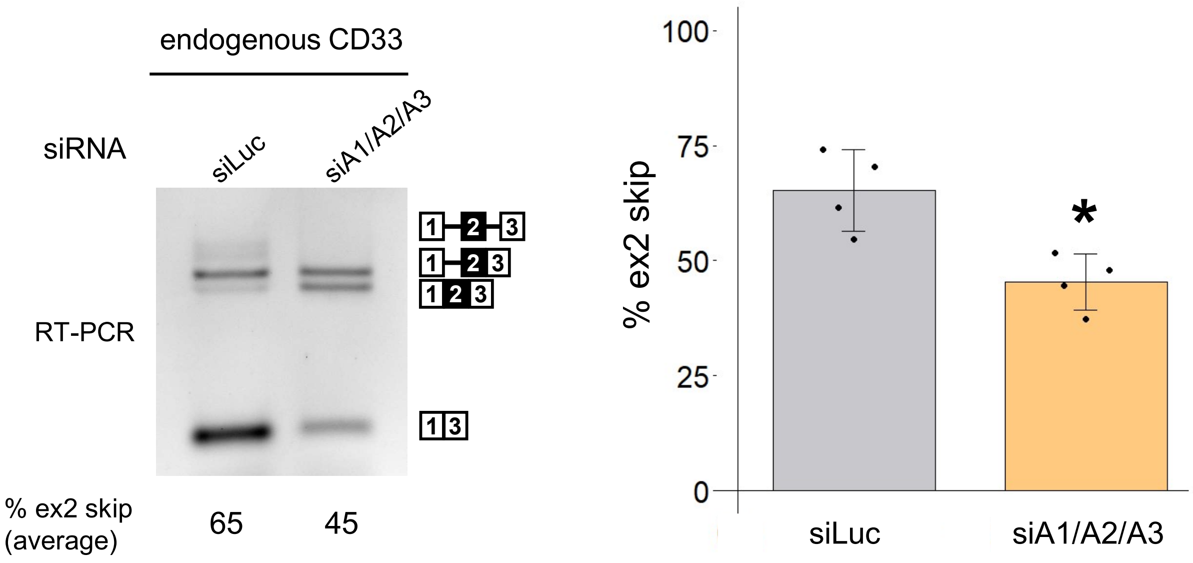

Figure S4. *CD33* splicing regulation by HNRNPA proteins in human microglial-like cells.
Quantitative analysis (right panel) of human induced pluripotent stem cell-derived microglia-like cells (iCell Microglia) treated with siRNA and subjected to the *CD33* splicing assay using RT-PCR (left panel). Error bars represent the SD (n = 4). Welch's t-test was used for statistical evaluation (*p = 0.0102).

Figure S5

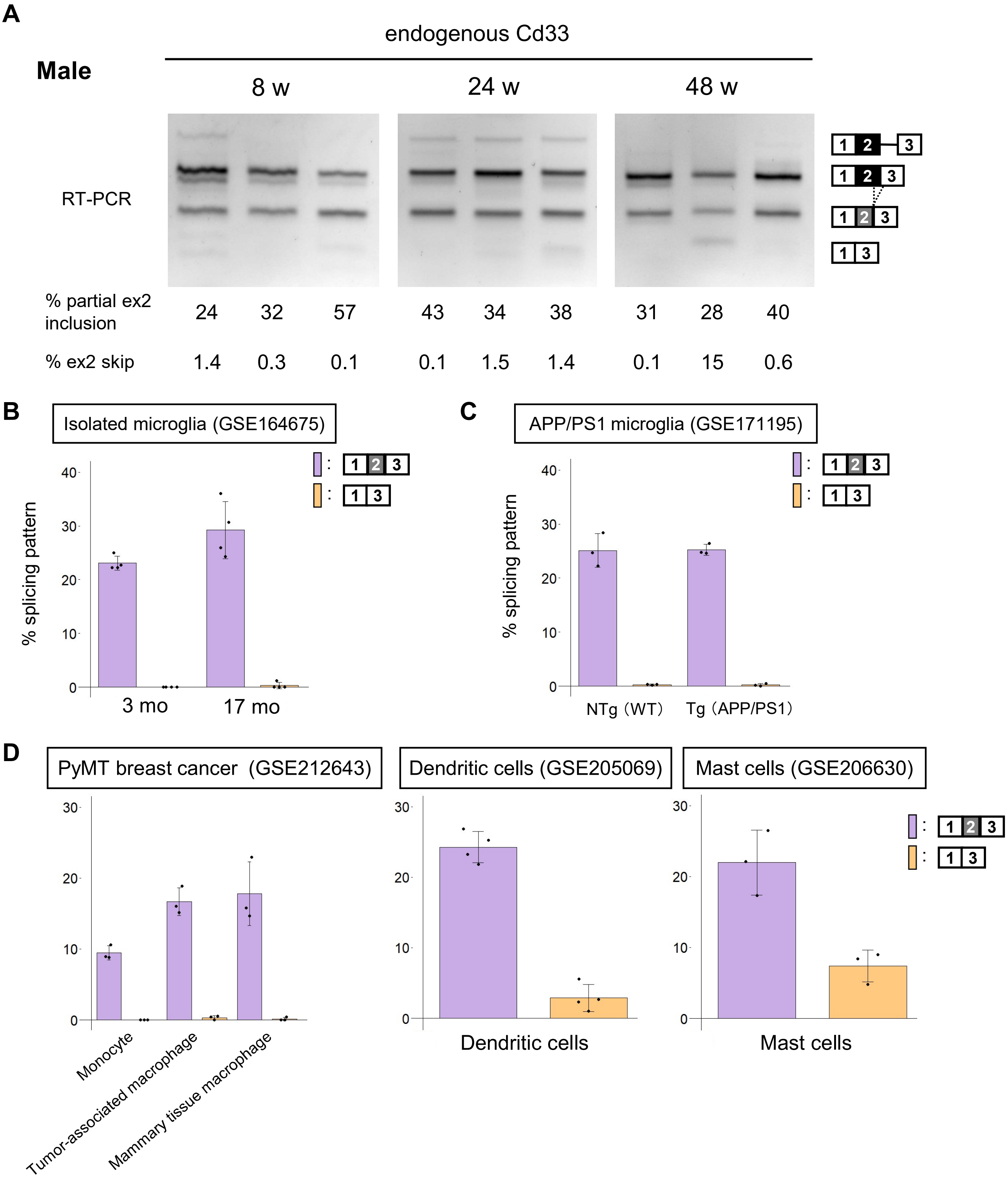

Figure S5. Alternative splicing of mouse *Cd33* exon 2.

(**A**) Splicing assay of mouse *Cd33* in the hippocampus of male mice of different ages.

(**B**) Splicing analysis of Cd33 using RNA-seq data (GSE164675) of microglial cells isolated from 3 and 17 months old mice (n = 4).

(**C**) Splicing analysis of Cd33 using RNA-seq data (GSE171195) of microglial cells isolated from transgenic Alzheimer's disease model male mice and non-transgenic control male mice at 10 months old (n = 3).

(**D**) Splicing analysis of Cd33 using RNA-seq data of myeloid cells from PyMT breast cancer model mice (n = 3, female at unspecified age, GSE212643), dendritic cells (n = 4, from 6–8 weeks old female mice, GSE205069), and Mast Cells (n = 3, from bone mallow of BALB/c mice, GSE206630). In B-D, error bars represent SD.

Figure S6

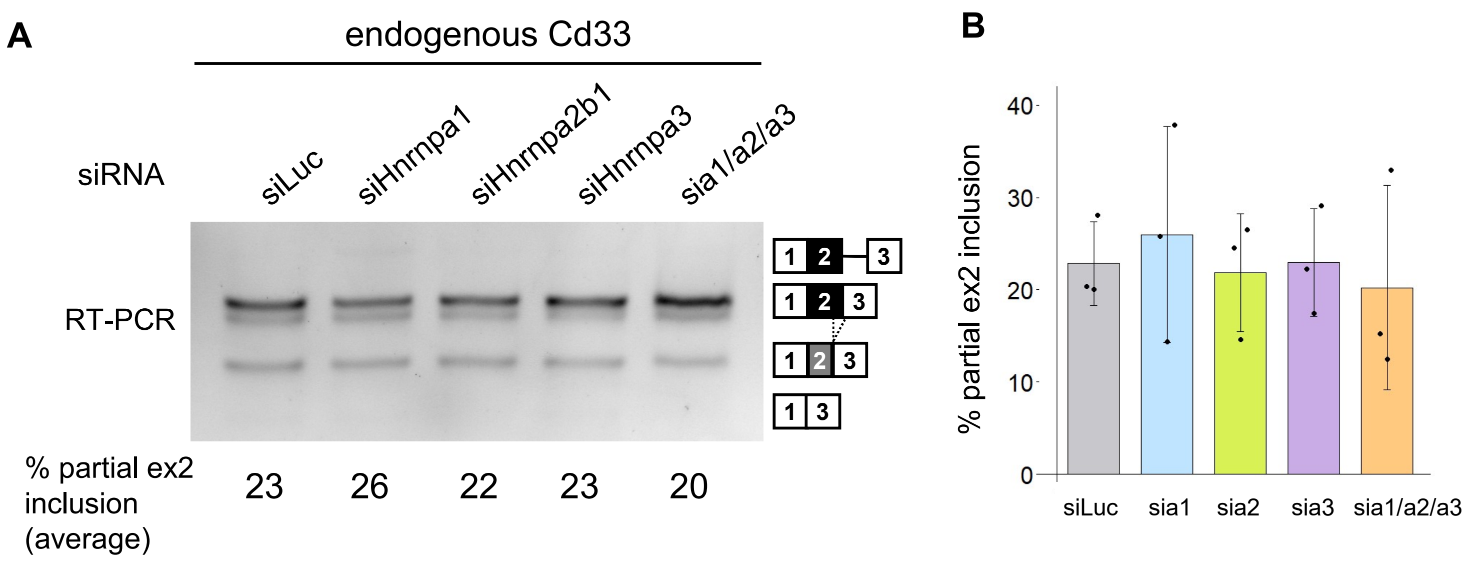

Figure S6. Effect of siRNA targeting HNRNPA proteins on *Cd33* splicing in RAW264.7 cells.

(**A**) RAW264.7 cells were treated with siRNA and analyzed using *Cd33* splicing assay. There was negligible detection of exon 2 skipping.

(**B**) Quantification of splicing assay results. Bar chart shows the proportion of partial exon 2 inclusion (n = 3). Error bars represent SD. No significant difference was observed (Tukey's test).

Figure S7

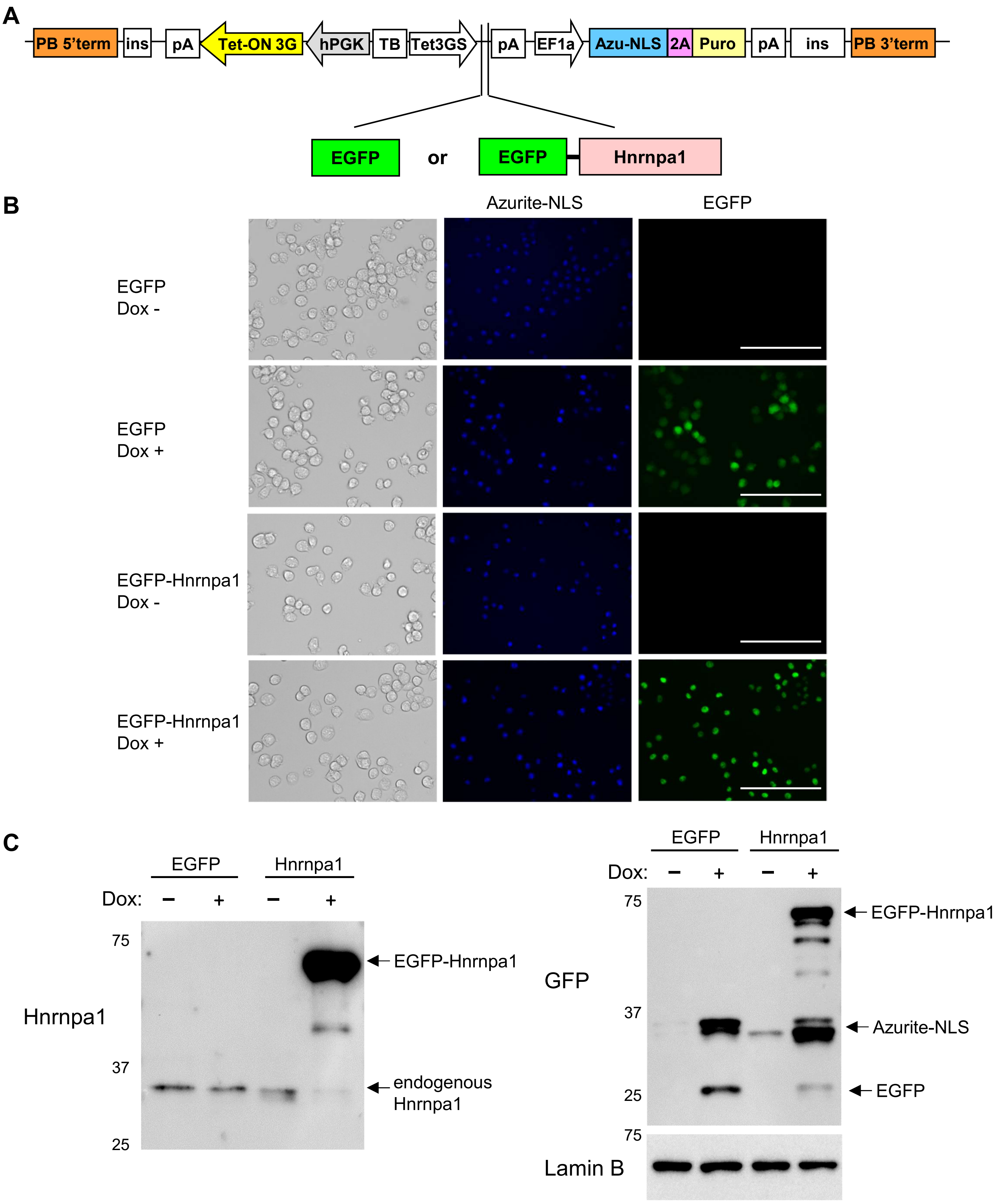

Figure S7. RAW264.7-based cells inducibly expressing EGFP or EGFP-Hnrnpa1.

(**A**) PB-Tet-Azu-Puro vector used for inducible expression of EGFP and EGFP-Hnrnpa1. Azu: Azurite blue fluorescence protein fused with three copies of nuclear localization signal. Structure details are as in Fig. S3A.

(**B**) Expression of Azu and EGFP-Hnrnpa1 in the RAW264.7 cells harboring the above constructs. Cell images in the presence and absence of doxycycline are shown. Azu was visualized using DAPI filter panels. Scale bar: 150 μm.

(**C**) Expression of EGFP and EGFP-Hnrnpa1 detected with western blotting using indicated antibodies.

Figure S8

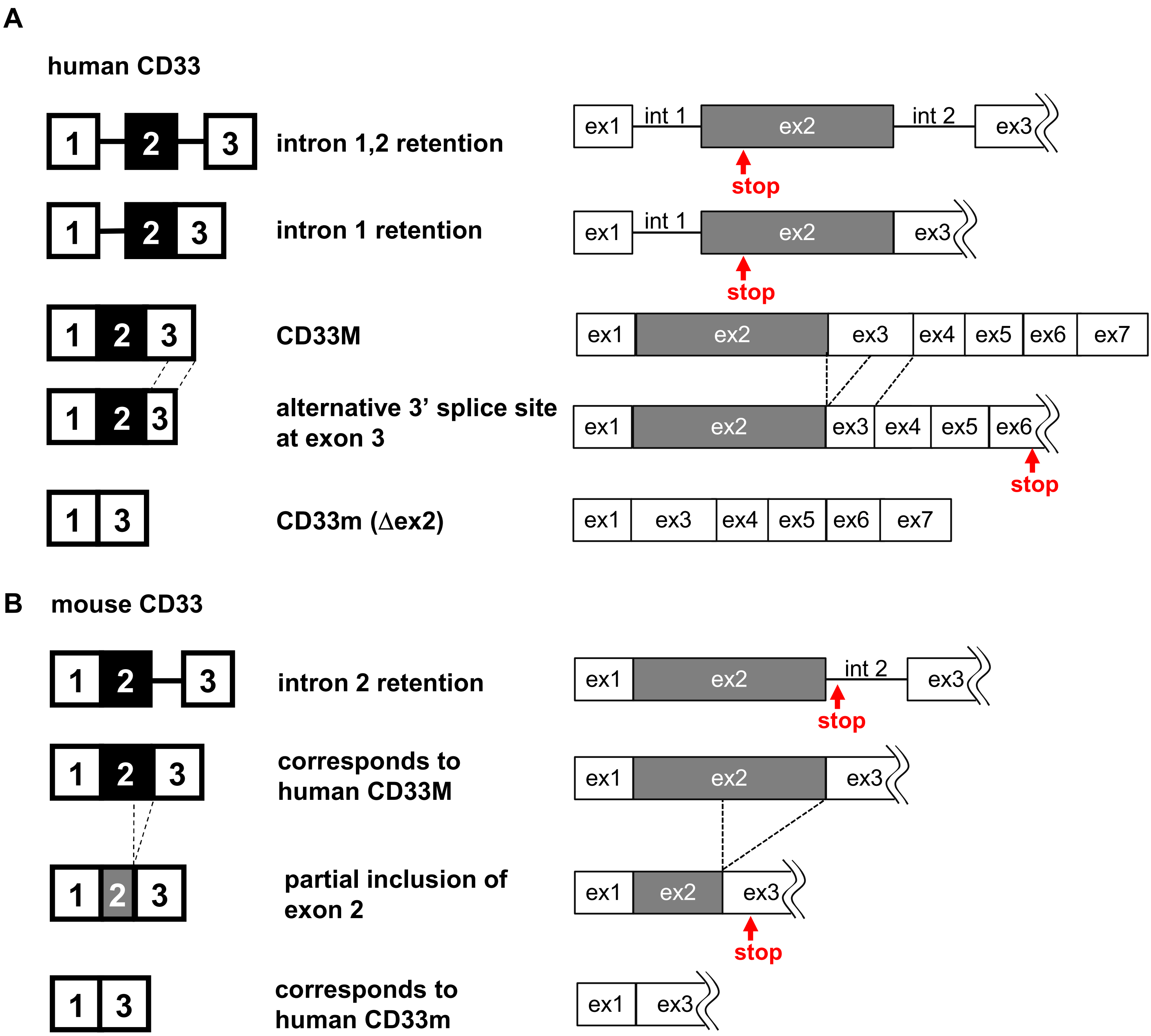

Figure S8. Prediction of CD33 proteins derived from the detected splicing products. Termination codons that lead to truncation of the C-terminus are shown in red.

Figure S9

Fig. 1B

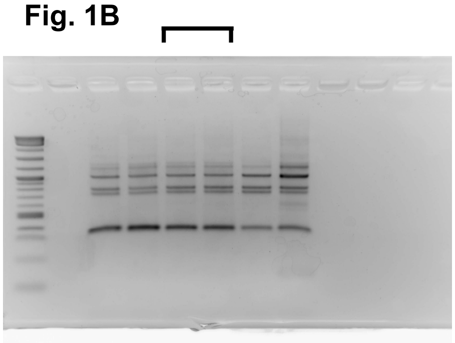

Fig. 1D

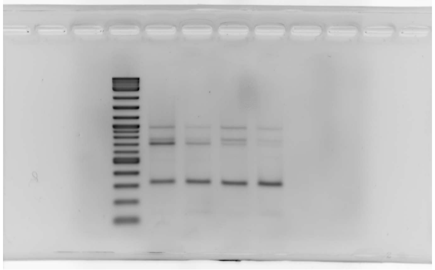

Fig. 1C

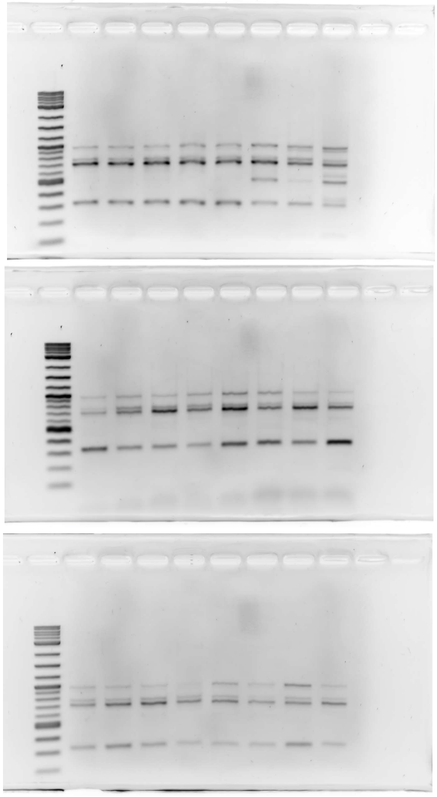

Fig. 2A

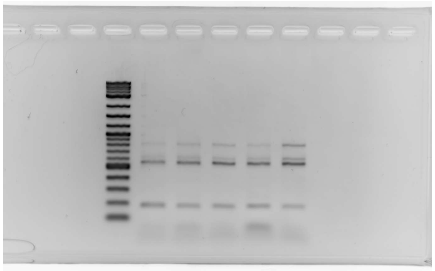

Fig. 2B

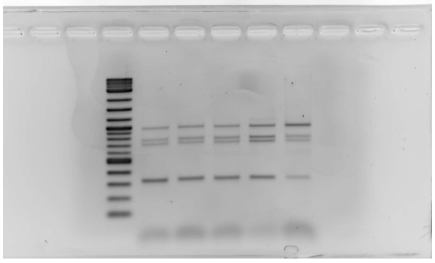

Fig. 2C

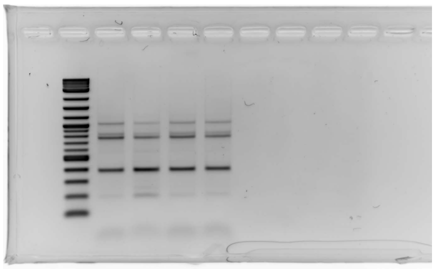

Fig. 3A

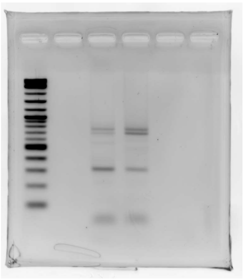

Fig. 5A

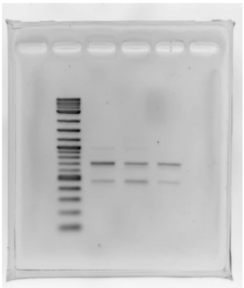

8 w

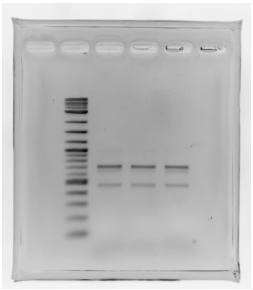

24 w

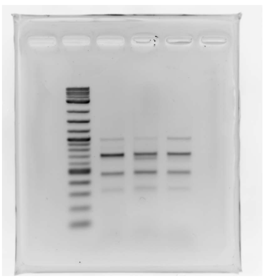

48 w

Fig. 3B

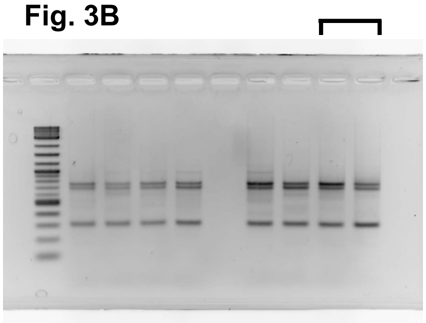

Fig. 3C

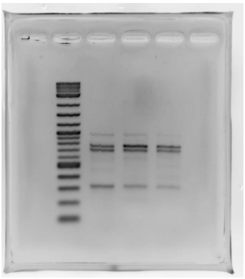

Fig. 4A

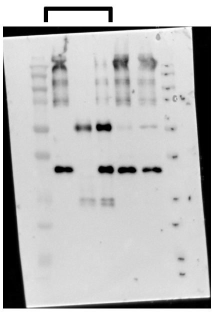

Fig. 6B

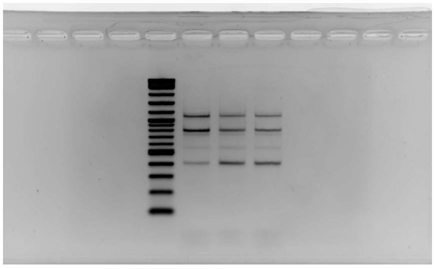

Fig. 6C

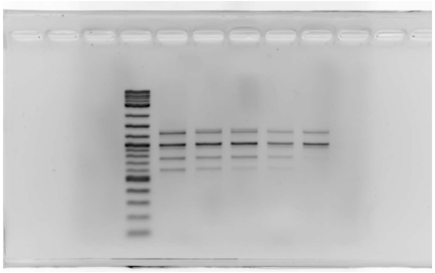

Fig. 6D

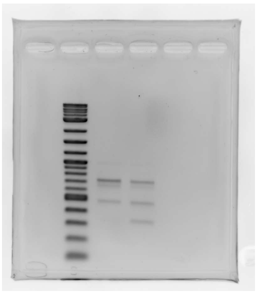

Figure S9. Uncropped images.

Figure S9 (continued)

Fig. S2A

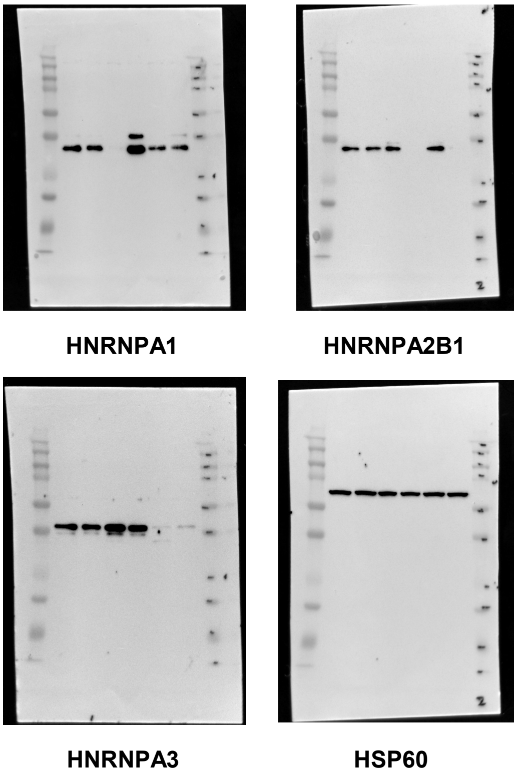

Fig. S2B

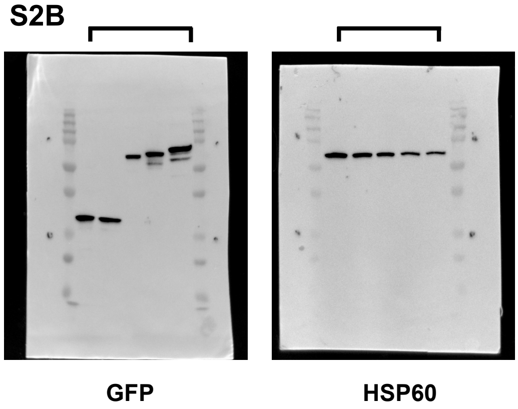

Fig. S2C

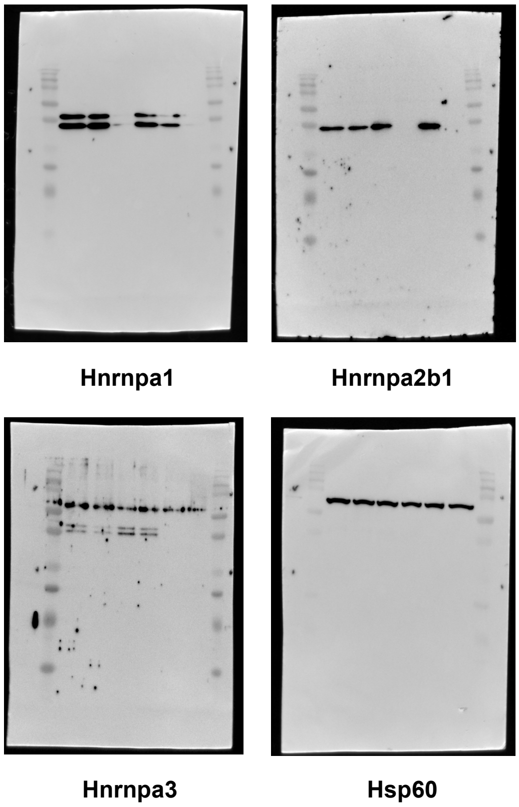

Fig. S3C

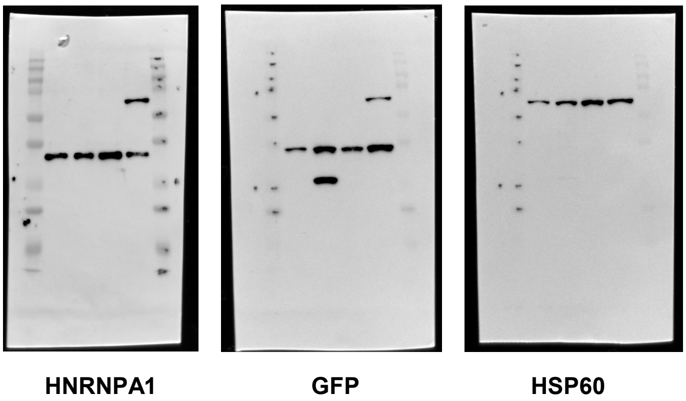

Fig. S4

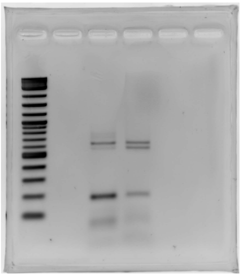

Fig. S5

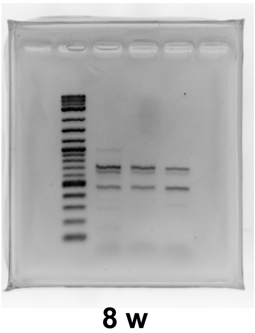

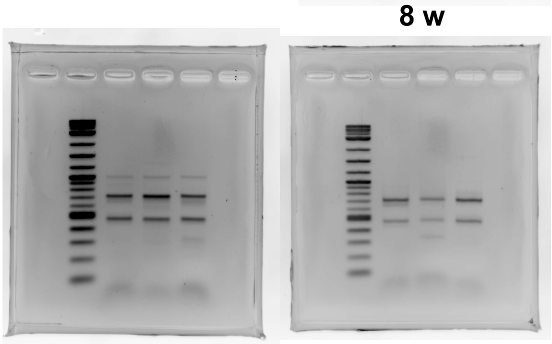

Fig. S6

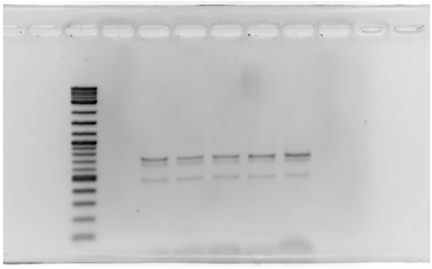

Fig. S7C

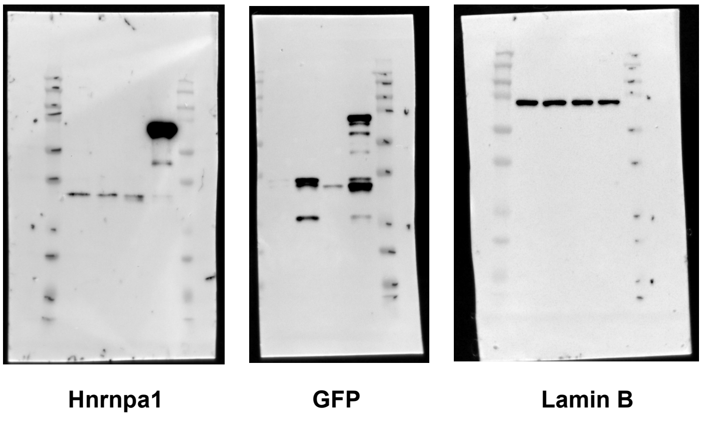

Figure S9. Uncropped images.

Table S1. List of oligonucleotides used in this study

Primers for constuction

**Construct**	**Primer**	**Sequence (5' to 3')**
CD33 minigene	BglII-CD33-ex1-Fw	AAAAAGATCTATGCCGCTGCTGCTACTGCTGCCCC
	XhoI-CD33-ex3-Rv	AAAACTCGAGAGGTGACGTTGAGCTGGATGGTTCT
	nest-CD33-Fw	CTGAGGTCTCCTGCTCCTCCCCAGCTTCCTGTCCG
	nest-CD33-int3-Rv	GTCAAGCTCCCCCGCTAAGCACGACAGGCTTTACC
	CD33-A14V-Fw	TCCCCACAGGGGTCCTGGCTATGGATCCAAATTTC
	CD33-A14V-Rv	TCCATAGCCAGGACCCCTGTGGGGAAACGAGGGTC
mouse Cd33 minigene	nest-moCd33-Fw	ACCCATGACAGGAAGCGTCAACCACCCAAC
	nest-moCd33-Rv	AACTAGTCAAGACATCATAGTCAGAAAGAC
	BglII-moCd33-ex1-Fw	AAAAAAGATCTATGCTGTGGCCACTGCCGCTGTTC
	XhoI-moCd33-ex3-Rv	AAAAACTCGAGGGGTAACATTGAGCTGGATGGTC
human hnRNPA1	nest-huHNRNPA1-Fw	CGCCGAAGAAGCATCGTTAAAGTCTCTCTTCACCC
	nest-huHNRNPA1-Rv	TGTCACTTCTCTGGCTCTCCTCTCCTGCTAAGCTT
	BglII-huHNRNPA1-Fw	AAAAAGATCTATGTCTAAGTCAGAGTCTCCTAAAG
	SalI-huHNRNPA1-Rv	AAAAAGTCGACTTAAAATCTTCTGCCACTGCCATA
OriCiro	SV40pA-oric-Fw	TGGTTTGTCCAAACTCATCAATGTATCTTAACGCGTAAATCTGCTCTGATGCCGCATAG
	CMV-oric-Rv	ATGAACTAATGACCCCGTAATTGATTACTATTAATAACTAGTGTCGGGGCTGGCTTAAC
	CMV-upstream-Fw	TAGTTATTAATAGTAATCAATTACGGGGTCATTAG
	SV40-downstream-Rv	ATTTACGCGTTAAGATACATTGATGAGTTTGGACA
mouse Hnrnpa1	BamHI-hnRNPA1-Fw	AAAGGATCCATGTCTAAGTCCGAGTCTCC
	XhoI-hnRNPA1-stop-Rv	AAACTCGAGTTAGAACCTCCTGCCACTG
Azurite	Azu-g197-Fw	TCGTGACCACCCTGAGCCACGGCGTGCAGTG
	Azu-g197-Rv	CACTGCACGCCGTGGCTCAGGGTGGTCACGA
	Azu-a451-Fw	TTCAACAGCCACAACATCTATATCATGGCCG
	Azu-a451-Rv	CGGCCATGATATAGATGTTGTGGCTGTTGAA
	BspEI-Azu-Rv	AAAAATCCGGACTTGTACAGCTCGTCCATGCCGTGAGTGATCCCGGCGGCGGTCCTGAACTCCAG
PB-Tet vector	NheI-Tet-Fw	AAAAAGCTAGCCCATCACTAGGGGTTCCTAGATCT
	SalI-Tet-Rv	AAAAAAGTCGACCACTAGGGGTTCCTATCGATATC
	BamHI-EGFP-Rv	AAAAAGGATCCTCAGAGTCCGGACTTGTACAGCTC
	CMV-Fw2-new	TCCAAGTCTCCACCCCA
	SV40pA-seq-Rv	AATGCTTTATTTGTGTTGTGATGCTATTGC

Primers for splicing assay

**Target**	**Primer**	**Sequence (5' to 3')**
human CD33 minigene	EGFP-C1-Fw	CATGGTCCTGCTGGAGTTCGTG
	CD33-ex3-Rv	GACGTTGAGCTGGATTCTCCGTAGTCACAC
	CD33-ex3-52-Rv	GCAGGTCAGGTTTTTGGAGT
	CD33-ex1-Fw	GAAGCTGCTTCCTCAGCCGCTGCTGCTACT
endogenous human CD33	CD33-new-Fw2	TCAGACATGCCGCTGCT
	CD33-new-Rv2	CTGGATGGTTCTCTCCGTAGTC
	CD33-new-Fw5	CTGCTTCCTCAGACATGCCG
	CD33-new-Rv5	CAGGAGAAGATCGGGGGTGT
mouse Cd33 minigene	EGFP-C1-Fw	CATGGTCCTGCTGGAGTTCGTG
	XhoI-moCd33-ex3-Rv	AAAAACTCGAGGGGTAACATTGAGCTGGATGGTC
	SV40pA-seq-Rv	AATGCTTTATTTGTGTTGTGATGCTATTGC
mouse endogenous Cd33	Cd33-ex1-splicing-Fw	CCACTGCCGCTGTTCTT
	moCd33-ex4-sp-Rv2	CTCTCTCATCTGGCCTGATTTC
	XhoI-moCd33-ex3-Rv	AAAAACTCGAGGGGTAACATTGAGCTGGATGGTC

Primer pairs used for splicing assay

Figure	Primer	Target
Fig. 1B,C,D, 2B,C	CD33-ex1-Fw	HEK293T human CD33 CV&A14V minigenes (over expression)
	CD33-ex3-Rv	
Fig. 2A	EGFP-C1-Fw	HEK293T human CD33 CV minigene (RNAi)
	CD33-ex3-52-Rv	
Fig. 3A,B	CD33-new-Fw2	THP-1 endogenous CD33
	CD33-new-Rv2	
Fig. 3C	CD33-new-Fw2	Cellartis Microglia endogenous CD33
	CD33-new-Rv2	
Fig. 5A	Cd33-ex1-splicing-Fw	mouse hippocampus endogenous Cd33
	XhoI-moCd33-ex3-Rv	
Fig. 6B	EGFP-C1-Fw	Cd33 minigene (HNRNPA1/Hnrnpa1 overexpression in HEK293)
	XhoI-moCd33-ex3-Rv	
Fig. 6C	EGFP-C1-Fw	Cd33 minigene (RNAi in Neuro2a)
	SV40pA-seq-Rv	
Fig. 6D, S5	Cd33-ex1-splicing-Fw	RAW264.7 endogenous Cd33 (A1 over expression)
	moCd33-ex4-sp-Rv2	
Fig. S4	CD33-new-Fw5	iCell Microglia endogenous CD33
	CD33-new-Rv5	

siRNA

Target	Sense strand [19 mer(RNA) + TT]
siHNRNPA1-440	gcuuugccuuuguaaccuuTT
siHNRNPA2B1-841	uuccacuuccauaauuuccTT
siHNRNPA3-1105	ggaaguggguggauaugguaTT
siHnrnpa1-167	gcuuuggguuugucacauaTT
siHnrnpa2b1-30	gcucuuuauugguggcuuaTT
siHnrnpa3-564	ccacacuauuaaugggcauTT

qPCR primers for RIP

Target	Primer	Sequence (5' to 3')
CD33 intron 2	RT-CD33-610-Fw6	CAGAGGTTGATCTTCTCTCAGG
	RT-CD33-679-Rv6	CCTGTGGGTCAAGTCTAGTG
CD33 exon 7	RT-CD33-ex7-Fw	TACTGTGGAGATGGATGAGGAG
	RT-CD33-ex7-Rv	GGGTCCTGACCTCTGAGTATT

Table S2. List of antibodies used in this study

primary antibodies	Source	Identifier	Dilution
mouse monoclonal Anti-hnRNP A1	abcam	9H10	1:200
mouse monoclonal Anti-hnRNP A1	Biolegend	4B10	1:30 (IP)
mouse monoclonal anti-hnRNP A2B1	abcam	DP3B3,ab6102	1:1000
rabbit polyclonal anti-HNRNPA3	proteintech	25142-1-AP	1:1000
goat polyclonal anti-Lamin B	santa cruz	sc-6217,M-20	1:3000
goat polyclonal anti-HSP60	santa cruz	sc-1052, N-20	1:1000
mouse monoclonal anti-GFP	santa cruz	sc-9996, B-2	1:3000

secondary antibodies	Source	Identifier	Dilution
Donkey Anti-Goat IgG H&L (HRP)	abcam	ab97120	1:5000
Goat Anti-Mouse IgG H&L (HRP)	abcam	ab97040	1:5000
Goat Anti-Rabbit IgG H&L (HRP)	abcam	ab6721	1:5000

Table S3. List of RNA-seq data used for splicing analysis

Figure	GEO ID	BioProject ID	Sample source	Preparation	Experiments
Fig. S5B	GSE164675	PRJNA691543	3 mo microglia	Isolated from brain tissues using Cd11b+ magnetic microbeads	SRX9833337, SRX9833338, SRX9833339, SRX9833340
			17 mo microglia		SRX9833349, SRX9833350, SRX9833351, SRX9833352
Fig. S5C	GSE171195	PRJNA718619	Ntg (WT)	Isolated from hippocampi using FACS (CD45lo/CD11b+ cells)	SRX10484534, SRX10484535, SRX10484539
			Tg (APP/PS1)		SRX10484529, SRX10484540, SRX10484541
Fig. S5D	GSE212643	PRJNA876305	Monocyte	Isolated from PyTM mouse tissues using FACS	SRX17415932, SRX17415931, SRX17415930
			Tumor-associated macrophage		SRX17415926, SRX17415925, SRX17415924
			Mammary tissue macrophage		SRX17415929, SRX17415928, SRX17415927
Fig. S5D	GSE205069	PRJNA843611	Dendritic cell	Cell Trace Violet-stained BMDC isolated using FACS	SRX15498788, SRX15498787, SRX15498786, SRX15498785
Fig. S5D	GSE206630	PRJNA851610	Mast cell	Primary cultured cells from mouse femurs	SRX15823347, SRX15823346, SRX15823345
